# Real-World Outcomes of CDK4/6 Inhibitors Treatment in Metastatic Breast Cancer in Romania

**DOI:** 10.3390/diagnostics13111938

**Published:** 2023-06-01

**Authors:** Andreea-Iuliana Miron, Alexandra-Valentina Anghel, Andrei-Alexandru Barnonschi, Ruxandra Mitre, Horia-Dan Liscu, Estera Găinariu, Raluca Pătru, Simona Coniac

**Affiliations:** 1 Department of Oncological Radiotherapy and Medical Imaging, “Carol Davila” University of Medicine and Pharmacy, 020021 Bucharest, Romania; andreea-iuliana.miron@drd.umfcd.ro (A.-I.M.);; 2 Department of Medical Oncology, Colțea Clinical Hospital, 030167 Bucharest, Romania; 3 Department of Radiotherapy, Colțea Clinical Hospital, 030167 Bucharest, Romania

**Keywords:** CDK 4/6 inhibitors, metastatic breast cancer HR+, progression-free survival, real-world data

## Abstract

The introduction in clinical practice of selective cyclin-dependent kinase (CDK) 4/6 inhibitors improves the outcome of patients with hormone receptor (HR)-positive human epidermal growth factor receptor 2 (HER2)-negative metastatic breast cancer (mBC). In Romania, the three available CDK 4/6 inhibitors (Palbociclib, Ribociclib and Ademaciclib) have been approved by the National Agency for Medicines (ANM) in 2019, 2020 and 2021. We conducted a retrospective study from 2019 to 2022 on 107 patients with metastatic breast cancer HR+ that have been treated with CDK 4/6 inhibitors in addition to hormone therapy in the Oncology Department of Colțea Clinical Hospital in Bucharest. The purpose of this study is to calculate the median progression-free survival (PFS) and to compare it with the median PFS from other randomized clinical trials. A key difference from other studies is that our study evaluated both patients with non-visceral mBC and patients with visceral mBC, as these two groups often have different outcomes. A total of 79.4% were postmenopausal patients and 20.6% were premenopausal; 42.1% had different stages at the beginning of disease and 57.9% presented newly metastatic disease. Median PFS was 17 months, unlike randomized clinical trials which reported a median PFS of 25.3 months. The combination of CDK 4/6 inhibitors with endocrine therapy is the golden standard treatment in HR-positive, HER2-negative metastatic breast cancer, bringing a prolongation of survival for these patients. Our results show no major differences compared to randomized clinical trials, despite the smaller patient group. In order to have a picture of the efficacy of the treatment as close as possible to the real-world data, we believe that it would be very useful to have a collaboration between several oncology departments in different institutions to carry out a multi-center study on large groups of patients.

## 1. Introduction

Breast cancer is the most common malignancy diagnosis at a global level, accounting for more than 2 million cases each year [1]. It is also the leading cause of cancer death in women worldwide, according to Globocan 2020 [2].

Stage IV breast cancer, either de novo at presentation or secondary after a primary diagnosis, is a heterogeneous disease. Although patients diagnosed with metastatic breast cancer (mBC) are unlikely to be cured, systemic therapies, hormone therapies and targeted therapies have contributed to a significant improvement in overall survival and progression-free survival over the past decade, especially among those with positive hormone receptor and negative HER growth factor overexpression [3,4,5,6]. A survival of 18 to 36 months has been reported for patients with mBC in different studies, although this range extends from a few months to several years. This gap may vary depending on the availability of effective therapies and the patient’s age, performance status, comorbidities, tumor subtype, disease extension, number of metastatic sites and locations [7,8,9]. Over the past decade, the median survival of patients with mBC has improved, a trend that has been attributed to the availability of new agents including taxanes, aromatase inhibitors, CDK 4/6 inhibitors, pertuzumab, trastuzumab and human epidermal growth factor (HER2) targeting agents [10,11,12,13].

Inhibition of cyclin-dependent kinase 4 (CDK4) and cyclin-dependent kinase 6 (CDK6) has shown considerable promise in attenuating resistance to endocrine therapy [14,15]. CDK4 and CDK6 are not only essential for G1 to S phase cell cycle transition, but also play a central role in the growth of HR+ breast cancer cells [16,17,18,19]. Thus, in clinical practice, inhibition of CDK4 and CDK6 has been an effective way of treating advanced HR+, HER2- breast cancer.

Oral CDK4/6 inhibitors have been shown to increase response rates and prolong disease control when combined with endocrine therapy in HER2-negative hormone-responsive (HR+) metastatic breast cancer. In Romania, the three available CDK 4/6 inhibitors (Palbociclib, Ribociclib and Abemaciclib) have been approved by the National Agency for Medicines (ANM) in 2019, 2020 and 2021, respectively, in combination with nonsteroidal aromatase inhibitors in first-line therapy for postmenopausal women, with a 40–45% improvement in progression-free survival. Additional approved indications include first- and second-line combination therapy for premenopausal women, combination with Fulvestrant and use as monotherapy, varying by agent. CDK4/6 inhibitors differ in toxicity profiles and monitoring requirements, and prescribers should be aware of the individual requirements for each agent.

In terms of duration of treatment, unlike the adjuvant situation, there is no predetermined duration of treatment. Therefore, the duration of therapy must be individualized, taking into account the patient’s treatment objectives, response to the disease, the presence of side effects and alternative options that may be available. In general, patients should continue treatment until disease progression or unacceptable toxicities occur. In our practice, we monitor treatment failure¸ taking into account changes in symptoms, physical examination, tumor markers and evidence of disease progression based on imaging.

## 2. Materials and Methods

This study was conducted in the Medical Oncology Clinic of Colțea Clinical Hospital Bucharest and included 107 patients with HR+ HER2- metastatic breast cancer who received CDK4/6 inhibitor treatment in combination with hormone therapy from January 2019 to March 2023. There were 58 patients in treatment with Palbociclib, 15 with Abemaciclib and 34 with Ribociclib, together with hormone therapy. Patients had an Eastern Cooperative Oncology Group (ECOG) performance status between 0–3 (on a 5-point scale where higher scores reflect greater disability). Adverse reactions were monitored and classified according to the CTCAE Common Terminology Criteria for Adverse Events (version 4.0) [20].

The parameters studied were age, menopausal status and those predicting rate of progression and survival, including the duration of time from diagnosis to metastasis in months, distribution of the studied group on each CDK4/6 inhibitor, association of each CDK 4/6 inhibitor with hormone therapy, distribution of ECOG performance status per CDK4/6 inhibitor, number of metastatic sites, distribution of non-visceral and visceral metastases per CDK4/6 inhibitor, total death rate, distribution of death per inhibitor and progression-free survival.

The objective of this study was to compare the group treated in the Medical Oncology Department of the Colțea Clinical Hospital with the group included in the real-world data evaluations in terms of structure and outcomes.

### 2.1. Inclusion Criteria

Patients with histologically and/or cytologically confirmed HR+/HER2- mBC;

Patients aged over 18 years at the time of breast cancer diagnosis;

Patients with mBC without visceral crisis (as defined by ABC guidelines);

Patients with mBC in line I or subsequent lines.

### 2.2. Exclusion Criteria

Patients in visceral crisis.

### 2.3. Treatment

Treatment was administered until disease progression, unacceptable toxicity, death or discontinuation of treatment for any other reason.

Palbociclib was administered orally 125 mg per day—3 weeks on, 1 week off (3/1 schedule), along with endocrine therapy. The first dose reduction was to 100 mg/day, and the second dose reduction was to 75 mg/day. There were no changes of doses for the endocrine therapy [21].

Ribociclib was administered orally 600 mg (3 tablets of 200 mg) per day—3 weeks on, 1 week off, plus endocrine therapy. The first dose reduction was to 400 mg/day (2 tablets), and the second decrease was to 200 mg/day (1 tablet). Dose modifications of Ribociclib, were allowed to manage adverse events (AEs). No endocrine therapy dose changes were allowed [22].

Abemaciclib was administered on a continuous schedule (150 mg, twice daily) plus nonsteroidal aromatase inhibitor (1 mg Anastrozole or 2.5 mg Letrozole, daily, at the discretion of the treating physician). The first dose reduction was to 100 mg, twice daily, and the second decreased to 50 mg twice daily, while endocrine therapy did not suffer changes in dose [23]. 

### 2.4. Treatment Monitoring

In our clinic’s practice, we monitor treatment failure, taking into account serial changes in symptoms, physical examination or tumor markers and evidence of disease progression based on serial imaging examinations [24]. The criteria we used to define treatment failure include the following: clinical deterioration during treatment (i.e., increase in disease-associated symptoms, intolerable treatment toxicities, decreased performance status), evidence of new metastatic sites, increase in the size of previously documented metastatic lesions and interpretation by individual treating physicians, not using RECIST criteria similar to other clinical trials [13].

### 2.5. Study Limitations

This is an observational, retrospective study. Disease progression was based only on clinical and radiological assessment interpreted by individual clinicians, i.e., not using RECIST criteria similar to clinical trials. This study does not have a control arm consisting only of patients exclusively undergoing hormone therapy. Survival may also be affected by subsequent therapies in patients initially diagnosed with stages I/II/III.

Statistical analysis was made using IBM SPSS statistics software version 29.

## 3. Results

Out of 107 patients with HR+ HER2- metastatic breast cancer who were treated with CDK4/6 inhibitor along with hormone therapy at Coltea Clinical Hospital Bucharest, 58 patients were treated with Palbociclib, 15 with Abemaciclib, 34 with Ribociclib and the following parameters were studied: age, menopausal status, immunohistochemistry type and outcomes for each category.

**Figure 1:**
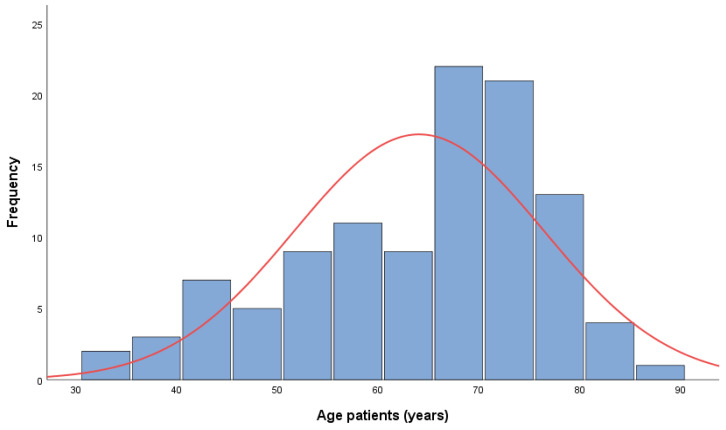
Age distribution.

As shown in Figure 1, The patients’ age ranged from 33 to 87 years, with a strong negative skewness (sk = −0.688) indicating a high proportion of patients over 64 years (CI95%: 61.69–66.44) (sd = 12.38); median value md = 67.0 years; and a quartile range Q1–Q3 (56–74). Approximately 75% of the patients were over 55 years of age.

From the frequency distribution of the age ranges presented in Table 1, negative skewness is observed, with the proportion of patients over 64 years being about 59%. The age interval with the lowest proportion is 18–49 years (15.0%), and the highest proportion is in the age interval 65–74 years (39.3%).

**Figure 2:**
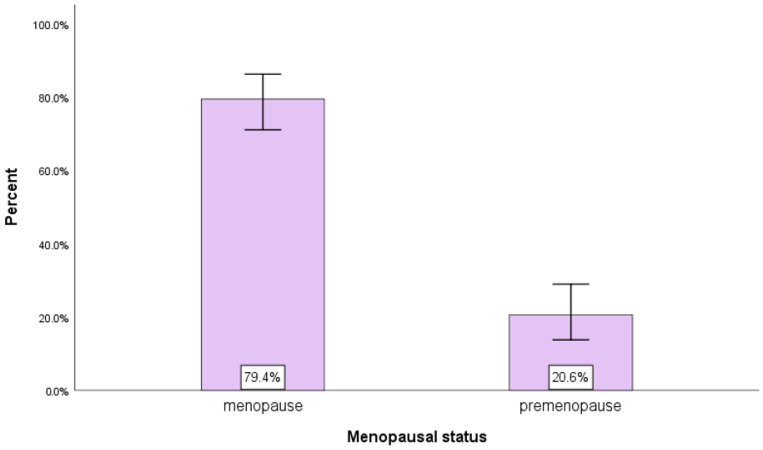
Menopausal status.

Figure 2 and Table 2 show that the proportion of patients with postmenopausal status is high (79.4%); the difference between the weights of the two status categories is statistically significant *p* < 0.001.

A high Luminal B proportion was observed (57.7%) in Table 3, but not statistically significantly different from the Luminal A weight for a test value of χ^2^(1) = 2.70 and *p* > 0.05.

Out of the total of 107 patients, 3 were initially luminal A then changed to luminal B due to disease mutations or failure of treatment appropriate to the category; thus, they were not included in the statistical analysis, leaving only 104 patients analyzed (Table 3).

As already well-known, cyclin-dependent kinase (CDK) activities coordinate the cell cycle progression through G1/S phase. However, different CDK activities and cyclins in driving cancer cell cycles are highly heterogeneous and can explain why some metastatic breast cancer patients can initially express high estrogen receptors, during which time they can turn into different expressions of estrogen/progesterone features, thus transforming from Luminal A to Luminal B. [25,26].

**Figure 3:**
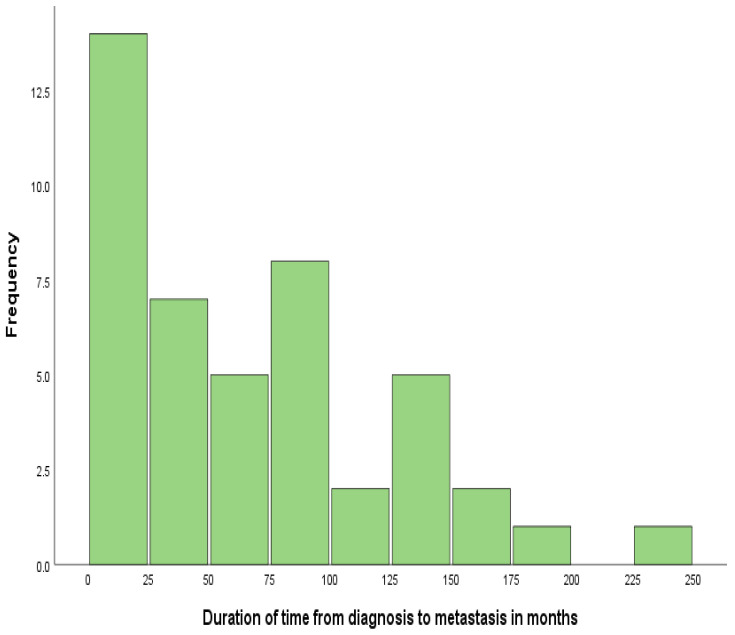
Duration in months from diagnosis to metastasis.

Of the total 107 patients, 57.9% (*n* = 62) patients were diagnosed as stage IV de novo, and the remaining 42.1% (*n* = 45) patients had stage II or III diagnosis. According to Figure 3, the time elapsed between diagnosis and first metastasis is a random variable that ranged from 1 to 232 months. The distribution shows a strong positive skewness with a high proportion of shorter durations when compared to the mean value (m = 68.0; sd = 58.27); median value md = 57.0 months; and a quartile range Q1–Q3 (20–103).

**Figure 4:**
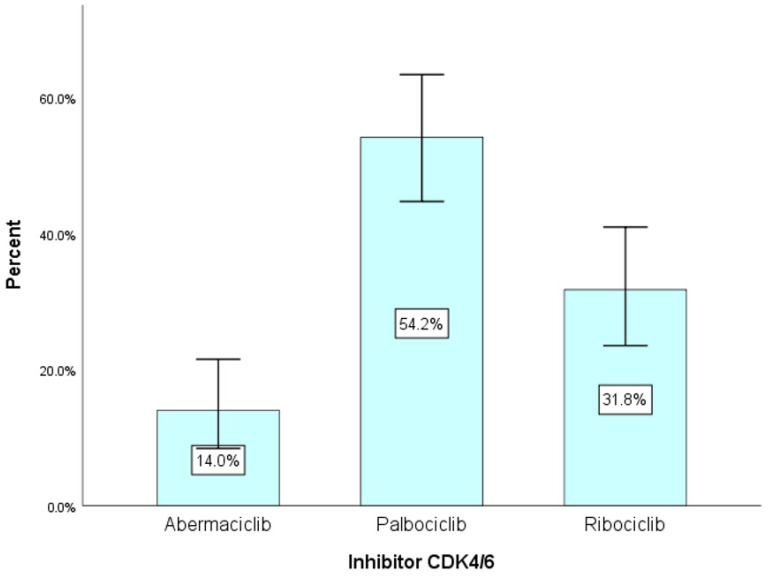
Distribution of patients per CDK inhibitor 4/6.

A low proportion of Abemaciclib inhibitor was observed (14%) according to Figure 4 and Table 4, while the highest proportion was observed for Palbociclib (54.2%), with a statistically significant difference χ^2^(2) = 26.04 and *p* < 0.001.

**Figure 5:**
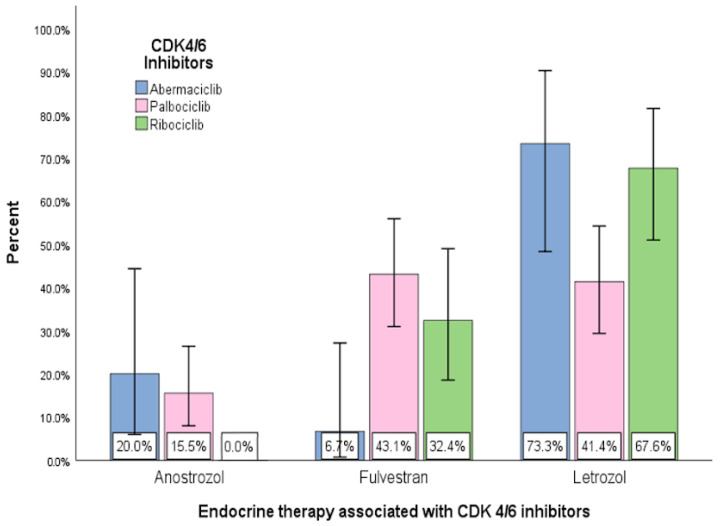
Combination of CDK 4/6 inhibitor (Palbociclib, Ribociclib, Abemaciclib) with endocrine therapy.

As shown in Table 5 and Figure 5, the most frequently used hormone therapy in our study is Letrozole (54.2%), followed by Fulvestrant (34.6%). Anostrozole was used in only about 11% of cases.

In the case of Fulvestrant, a statistically significant association difference was identified (χ^2^(4) = 14.36 with *p* < 0.01) with the type of CDK4/6 inhibitor used. Thus, in the case of Abemaciclib and Ribociclib, Letrozole was applied to a greater extent, with 73.3% for Abemaciclib and 67.6% for Ribociclib. In the case of Palbociclib, Fulvestrant (43.1%) and Letrozole (41.4%) were applied in approximately equal proportions.

**Figure 6:**
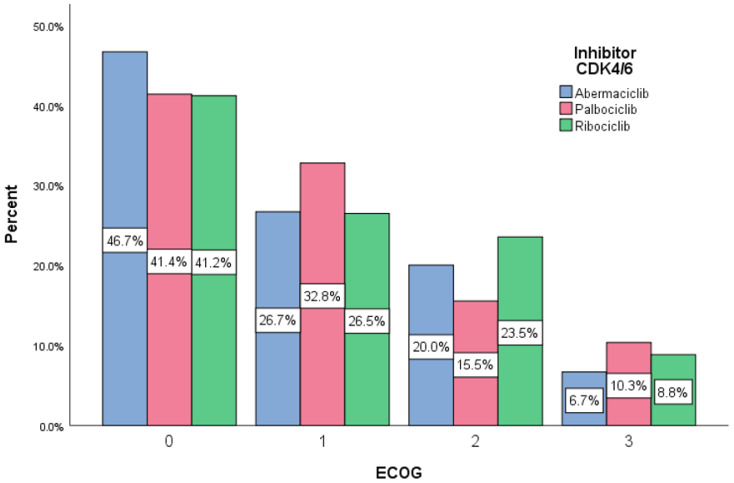
Distribution according to ECOG performance status.

Figure 6 indicates that the distribution of ECOG performance status is strongly skewed but correct from a medical point of view, with ECOG 0 in 42.1% cases, ECOG 1 in 29.9% cases, ECOG 2 in 18.7 cases and ECOG 3 being observed in only 9.3% cases.

In terms of the difference between the ECOG score distributions for the three types of CDK4/6 inhibitor, it was observed that they did not differ significantly (χ^2^(6) = 1.37.36 and *p* > 0.10).

A high proportion of stage IV (57.9%) was observed in Table 6, followed by stage II (23.4%) and stage III (18.7%). Between patient groups separated according to CDK 4/6 inhibitor type, the difference in disease stage is not statistically significant (χ^2^(4) = 3.34 and *p* > 0.10).

As shown in Table 7, of the 107 patients studied, 67.3% (*n* = 72) had visceral metastases and 73.6% (*n* = 78) had non-visceral metastases. For Palbocliclib patients, 69% (*n* = 40) had visceral metastases and 77.6% (*n* = 45) had non-visceral metastases. For Ribociclib patients (*n* = 34), 61.8% (*n* = 21) had visceral metastases and 76.5% (*n* = 26) had non-visceral metastases, while for Abemaciclib patients (*n* = 15), 73.3% (*n* = 11) had visceral metastases and 53.3% (*n* = 8) had non-visceral metastases.

As presented in Table 8, in terms of the number of metastatic sites, the highest proportion of patients had one metastatic site (37.4%) or two metastatic sites (30.8%). As many as 15% of cases had at least four metastatic sites.

An ordinal regression analysis was applied using the type of CDK4/6 inhibitor used as a factor. We used Abemaciclib as reference because it had the highest proportion (73.3%) of low numbers of metastatic sites (1–2).

As shown in Table 9, the ordinal logistic regression model does not reach statistical significance (W(2) = 3.51 and *p* > 0.10), but the odds ratio at the limit of statistical significance (W(1) = 2.89 *p* < 0.10) is 1.5 times higher for Palbociclib than for Abemaciclib (exp(B) = 1.50; CI95%: 0.94–2.38).

**Figure 7:**
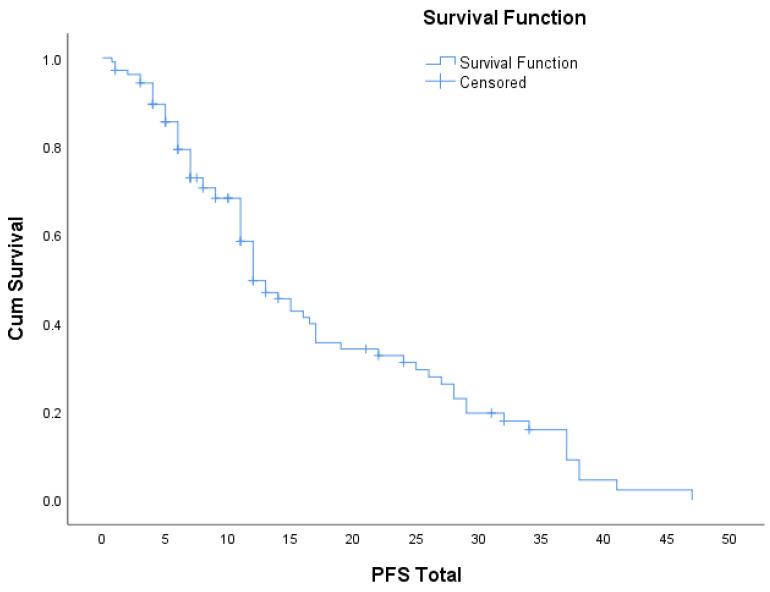
Progression-free survival.

In Table 10 and Figure 7, Kaplan–Meier analysis was applied for progression-free survival (PFS), considering the criterion event as lack of progression, resulting in a total PFS of 17.6 months.

**Figure 8:**
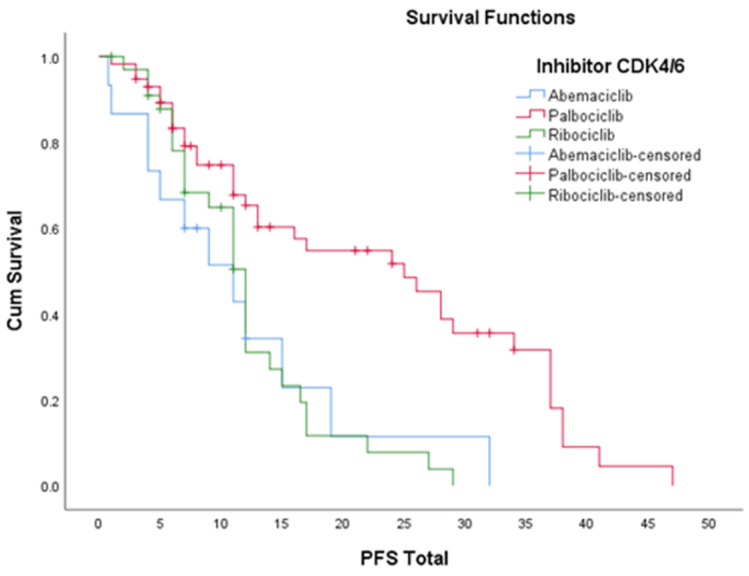
Progression-free survival on each CDK4/6 inhibitor.

As illustrated in Table 11 and Figure 8, the estimated PFS on each CDK4/6 inhibitor was uneven, so patients on Palbociclib (*n* = 58) had a mean PFS of 22.9 months, those on Ribociclib (*n* = 34) had a mean PFS of 12.06 months, and those on Abemaciclib (*n* = 15), 11.7 months. We consider that the inhomogeneous distribution of the number of patients on each of the three arms (patients on Palbociclib, patients on Ribociclib and patients on Abemaciclib) resulted in an inhomogeneous distribution of PFS.

Table 12 indicates that the death rate is low (22.4%). A binary logistic regression test was applied using CDK4/6 inhibitor type as a factor and Abemaciclib as reference.

According to Table 13, the model is not statistically significant and there was no statistically significant difference in the proportion of death depending on the type of CDK4/6 inhibitor used.

## 4. Discussion

There is only one ongoing real-world clinical trial investigating the efficacy of CDK4/6 inhibitor in metastatic breast cancer patients in Romania, with expected results in 2024 [27]. An updated search in PubMed of other daily practice analyses of inhibitor CDK4/6 treatment for metastatic breast cancer in Romania revealed just one case report, and no other published data are displayed in databases or registries [28]. Our real-world retrospective study is the first to show efficacy data on this research topic in Romania.

In terms of menopausal status, our group of patients has a high postmenopausal status of 79.4%, which is similar to what was reported in other real-world data studies [14,15].

In other real-world data studies, the patients had an Eastern Cooperative Oncology Group performance status between 0 and 1 (on a 5-point scale, where higher scores reflect higher disability); whereas, in our study, they were between 0 and 3. A proportion of about 72% had ECOG scores of 0 and 1 and only 9.3% of the patients had ECOG 3.

The distribution per CDK4/6 inhibitor was inhomogeneous, with Palbocliclib having the highest proportion of 54.2% and Abemaciclib having the lowest proportion of only 14%, with a statistically significant difference: χ^2^(2) = 26.04 and *p* < 0.001. According to www.fda.gov, the Food and Drug Administration approved Palbociclib in February 2015 as the first CDK4/6 inhibitor (in combination with Letrozole) for the treatment of HR-positive, HER2-negative advanced breast cancer as an initial endocrine based therapy in postmenopausal women. On 19 February 2016, the FDA approved Palbociclib in combination with Fulvestrant for the treatment of women with hormone receptor (HR)-positive and human epidermal growth factor receptor 2 (HER2)-negative advanced or metastatic breast cancer with disease progression following endocrine therapy [29]. In March 2017, the FDA also approved the second CDK4/6 inhibitor molecule in the treatment of HR + HER2- metastatic breast cancer—Ribociclib [30]. Six months later, in September 2017, the FDA approved the third molecule—Abemaciclib [31]. In Romania, the three molecules were approved much later, the first one in 2019 being Palbociclib, followed by Ribociclib in 2020, and Abemaciclib in 2021. The fragmented approval time of the three molecules made the distribution of each CDK4/6 inhibitor uneven [32,33,34,35,36].

The association of CDK 4/6 inhibitor with hormone therapy was in accordance with the literature. In line I of treatment, the PALOMA trials paired Palbociclib with Letrozole, the MONALEESA trials paired Ribociclib with Letrozole and the MONARCH trials paired Abemaciclib with Letrozole. In line II, these studies replaced Letrozole with Fulvestrant [28]. In our study, most patients were treated as per line I, adding as hormone therapy Letrozole (54.2%) + Anastrozole (11%); while line II treatment with Fulvestrant included 34.6% of patients.

In our study both patients who were diagnosed de novo in stage IV and those who received subsequent therapies received CDK4/6 inhibitors. Thus, at the time of diagnosis, we had 57.9% patients in stage IV, 23.4% patients in stage II, and 18.7% patients in stage III. Those in stage II and III at the time of diagnosis of breast cancer received subsequent therapies and, over time, progressed to metastatic stage and were given a CDK 4/6 inhibitor plus endocrine therapy.

In the PALOMA study, 60% of patients had visceral metastases and 40% non-visceral metastases. In our case, 69% (*n* = 40) of patients on Palbociclib had visceral metastases and 77.6% (*n* = 45) had non-visceral metastases. In the MONALEESA study, 42% of patients had visceral metastases, while for our Ribociclib arm (*n* = 34), 61.8% (*n* = 21) had visceral metastases and 76.5% (*n* = 26) had non-visceral metastases. The MONARCH study showed 52.7% patients with visceral metastases and 26.2% with non-visceral metastases, but our study on Abemaciclib treatment showed 73.3% (*n* = 11) of patients with visceral metastases and 53.3% (*n* = 8) with non-visceral metastases. Our study had a total of 107 patients of which 67.3% (*n* = 72) had visceral metastases and 73.6% (*n* = 78) had non-visceral metastases. Our data were similar to the aforementioned clinical trials in terms of the number of metastatic sites, with the highest proportions of patients having one and two metastatic sites (37.4% and 30.8%, respectively). The MONALEESA study divided the number of metastatic sites below three in 42% of cases and above three in 30% of cases.

In terms of progression-free survival in the overall population, the PALOMA study conducted on *n* = 347 patients treated with Palbociclib in combination with endocrine therapy showed a PFS of 34.9 months. Our study (*n* = 58), for the same combination of molecules, had a mean PFS of 22.9 months. The MONALEESA study had *n* = 484 patients treated with Ribociclib in combination with endocrine therapy and showed a PFS of 20.6 months. Our Ribociclib arm of 34 patients had a mean PFS of 12.06 months. The MONARCH study was conducted on *n* = 267 patients, obtaining a mean PFS of 27.3 months. We had very few patients on Abemaciclib in combination with endocrine therapy (*n* = 15) and obtained a mean PFS of 11.7 months. We consider that the uneven distribution of the number of patients on each of the three arms—Palbociclib, Ribociclib and Abemaciclib—led to an uneven distribution of PFS, and the much smaller number of patients in this study group compared to the number of patients in the real-world data led to a lower PFS [37,38,39,40,41,42].

## 5. Conclusions

The combination of CDK 4/6 inhibitors with endocrine therapy is the gold standard treatment in HR-positive, HER2-negative metastatic breast cancer, effectively prolonging survival for these patients.

Our results show no major differences compared to randomized clinical trials, despite the smaller patient group. In order to acquire a picture of the efficacy of the treatment as close as possible to the real-world data, we believe that it would be very useful to conduct a future collaboration between several oncology departments in different institutions to carry out a multicenter study on large groups of patients.

## Figures and Tables

**Table 1:** Age range distribution.

	Frequency	Percent	Cumulative Percent
18–49 years	16	15.0	15.0
50–64 years	28	26.2	41.1
65–74 years	42	39.3	80.4
>75 years	21	19.6	100.0
Total	107	100.0	

**Table 2:** Menopausal status.

Menopausal Status	Frequency	Percent	Cumulative Percent
Postmenopausal	85	79.4	79.4
Premenopausal	22	20.6	100.0
Total	107	100.0	

**Table 3:** Distribution according to immunohistochemistry—Luminal Type A/B.

IHC	Frequency	Percent	Cumulative Percent
Luminal A	44	42.3	42.3
Luminal B	60	57.7	100.0
Total	104	100.0	
Test Statistics			
Chi-Square	2.701		
Df	1		
Asymp. Sig.	0.100		

**Table 4:** Distribution of patients per CDK inhibitor 4/6.

	Frequency	Percent	Cumulative Percent
Abemaciclib	15	14.0	14.0
Palbociclib	58	54.2	68.2
Ribociclib	34	31.8	100
Total	107	100.0	

**Table 5:** Combination of CDK 4/6 inhibitor (Palbociclib, Ribociclib, Abemaciclib) with endocrine therapy.

CDK4/6 Inhibitors Endocrine Therapy Crosstabulation						
			Endocrine Therapy			Total
			Anostrozole	Fulvestrant	Letrozole	
CDK4/6 Inhibitors	Abemaciclib	15	3	1	11	15
		100.0%	20.0%	6.7%	73.3%	100.0%
	Palbociclib	58	9	25	24	58
		100.0%	15.5%	43.1%	41.4%	100.0%
			2.5	4.9	−7.4	
	Ribociclib	34	0	11	23	34
		100.0%	0.0%	32.4%	67.6%	100.0%
Total		Count	107	37	58	107
		%	100.0%	34.6%	54.2%	100.0%
Chi-Square Tests						
			Value	df	Asymptotic Significance (2-sided)	
Pearson Chi-Square			14.36	4	0.006	
Likelihood Ratio			19.46	4	0.001	
Linear-by-Linear Association			2.16	1	0.142	
N of Valid Cases			107			

**Table 6:** Disease stage at initial diagnosis.

Stage		Frequency		Percent		Cumulative Percent
IIA		17				
IIB		8		23.4		23.4
IIIA		7				
IIIB		11				
IIIC		2		18.7		42.1
IV		62		57.9		100
Total		107		100.0		
CDK4/6 Inhibitor STAGE Crosstabulation			Disease stage			Total
			II	III	IV	
InhibCDK4/6	Abemaciclib	Count	4	2	9	15
		%	26.7	13.3	60.0	100.0%
	Palbociclib	Count	10	13	35	58
		%	17.2	22.4	60.4	100.0%
	Ribociclib	Count	11	5	18	34
		%	32.4	14.7	52.9	100.0%
Total		Count	25	20	62	107
		%	23.4	18.7	57.9	100.0%
Chi-Square Tests		Value		df	Asymptotic Significance (2-sided)	
Pearson Chi-Square		3.34		4	0.502	
Likelihood Ratio		3.33		4	0.504	
N of Valid Cases		107				

**Table 7:** Distribution of metastasis according to site.

Visceral			Frequency	Percent		Cumulative Percent		
Yes			72	67.3		67.3		
No			35	32.7		100.0		
Non-visceral								
Yes			78	73.6		73.6		
No			28	26.4		100.0		
Total			104	100.0				
				Visceral Metastasis				Total
				No		Yes		
CDK4/6 Inhibitors	Abemaciclib		Count	4		11		15
			%	26.7%		73.3%		100.0%
	Palbociclib		Count	18		40		58
			%	31.0%		69.0%		100.0%
	Ribociclib		Count	13		21		34
			%	38.2%		61.8%		100.0%
Total			Count	35		72		107
			%	32.7%		67.3%		100.0%
Chi-Square Tests			Value	Df		Asymptotic Significance (2-sided)		
Pearson Chi-Square			0.794	2		0.672		
Likelihood Ratio			0.792	2		0.673		
N of Valid Cases			107					
					Non-visceral Metastasis		Total	
					No	Yes		
CDK4/6 Inhibitors		Abemaciclib		Count	6	8	14	
				%	46.7%	53.3%	100.0%	
		Palbociclib		Count	13	45	58	
				%	22.4%	77.6%	100.0%	
		Ribociclib		Count	8	26	34	
				%	23.5%	76.5%	100.0%	
Total				Count	28	78	106	
				%	26.4%	73.6%	100.0%	
Chi-Square Tests			Value	df		Asymptotic Significance (2-sided)		
Pearson Chi-Square			4.630	2		0.099		
Likelihood Ratio			4.167	2		0.124		
N of Valid Cases			106					

**Table 8:** Distribution according to the number of metastatic sites.

Nr of Sites		Frequency		Percent		Cumulative Percent	
1		40		37.4		37.4	
2		33		30.8		68.2	
3		18		16.8		85.0	
4–7		16		15.0		100.0	
Total		104		100.0			
Inhibitor CDK4/6 Nr of sites			Number of metastatic sites				Total
			1	2	3	4	
CDK4/6 Inhibitors	Abemaciclib	Count	9	2	3	1	15
		%	60.0%	13.3%	20.0%	6.7%	100.0%
	Palbociclib	Count	15	24	11	8	58
		%	25.9%	41.4%	19.0%	13.8%	100.0%
	Ribociclib	Count	16	7	4	7	34
		%	47.1%	20.6%	11.8%	20.6%	100.0%
Total		Count	40	33	18	16	107
		%	37.4%	30.8%	16.8%	15.0%	100.0%
Chi-Square Tests		Value	Df	Asymptotic Significance (2-sided)			
Pearson Chi-Square		11.930 ^a^	6	0.064			
Likelihood Ratio		12.310	6	0.055			
N of Valid Cases		107					

^a^ Three cells (25.0%) have expected count less than 5. The minimum expected count is 2.24.

**Table 9:** Ordinal logistic regression result.

Parameter	B	Std. Error	Hypothesis Test			Exp(B)	Model Efect
			Wald Chi-Square	df	Sig.		
(Intercept)	1.67	0.215	65.51	1	0.000	5.29	Wald Chi-square(2) = 3.51 *p* = 0.173
[Inhibitor CDK4/6 = 3]	0.19	0.25	0.54	1	0.462	1.21	
[Inhibitor CDK4/6 = 2]	0.40	0.24	2.89	1	0.089	1.50	
[Inhibitor CDK4/6 = 1]	0	.	.	.	.	1	

**Table 10:** Progression-free survival.

Means and Medians for Survival Time							
Mean ^a^				Median			
Estimate	Std. Error	95% Confidence Interval		Estimate	Std. Error	95% Confidence Interval	
		Lower Bound	Upper Bound			Lower Bound	Upper Bound
17.61	1.39	14.89	20.32	12.00	1.36	9.34	14.66

^a^ Estimation is limited to the largest survival time if it is censored.

**Table 11:** Progression-free survival on each CDK4/6 inhibitor.

Means and Medians for Survival Time								
CDK4/6 Inhibitors	Mean ^a^				Median			
	Estimate	Std. Error	95% Confidence Interval		Estimate	Std. Error	95% Confidence Interval	
			Lower Bound	Upper Bound			Lower Bound	Upper Bound
Abemaciclib	11.74	2.72	6.41	17.06	11.00	3.19	4.75	17.25
Palbociclib	22.90	2.16	18.67	27.12	25.00	5.06	15.09	34.91
Ribociclib	12.06	1.23	9.65	14.47	12.00	0.79	10.46	13.54
Overall	17.61	1.39	14.89	20.32	12.00	1.36	9.34	14.66

^a^ Estimation is limited to the largest survival time if it is censored.

**Table 12:** Death rate.

			Death		Total
			No	Yes	
CDK4/6 Inhibitors	Abemaciclib	Count	13	2	15
		%	86.7%	13.3%	100.0%
	Palbociclib	Count	42	16	58
		%	72.4%	27.6%	100.0%
	Ribociclib	Count	28	6	34
		%	82.4%	17.6%	100.0%
Total		Count	83	24	107
		%	77.6%	22.4%	100.0%
Chi-Square Tests		Value	Df	Asymptotic Significance (2-sided)	
Pearson Chi-Square		2.05	2	0.359	
Likelihood Ratio		2.12	2	0.347	
N of Valid Cases		107			

**Table 13:** Test of statistical significance.

Inhibitor	B	Std. Err.	Test			Exp(B)	CI 95% exp(B)	
			Wald	df	Sig.		Lower	Upper
Palbociclib	0.91	0.81	1.24	1	0.266	2.48	0.50	12.22
Ribociclib	0.33	0.88	0.14	1	0.707	1.39	0.25	7.86

## Data Availability

Data available on request due to ethical restrictions. The data presented in this study are available on request from the corresponding author and the Coltea Clinical Hospital (secretariat@coltea.ro). The data are not publicly available due to the policy of Coltea Clinical Hospital to have the approval of the Ethics Commitee for each new research study.
